# Yeast diversity in pit mud and related volatile compounds in fermented grains of chinese strong-flavour liquor

**DOI:** 10.1186/s13568-023-01562-7

**Published:** 2023-06-08

**Authors:** Yan Shoubao, Yang Jie, Shen TingTing, Guang Jiaquan, Shi Cuie

**Affiliations:** 1 Department of biology and food engineering, Huainan Normal University, Huainan, 230038 China; 2 Brewing Industry Microbial Resource Development and Application Engineering Research Center in Anhui Province, Huainan Normal University, Huainan, 230038 China; 3 Anhui Yingjia Group Co., Ltd, Luan, 237271 China

**Keywords:** Pit mud, Yeast, Volatile flavor compounds, Chinese strong flavour liquor, Fermentation

## Abstract

Chinese strong-flavour liquor is produced via a traditional solid-state fermentation strategy facilitated by live microorganisms in pit mud-based cellars. For the present analysis, pit mud samples from different spatial locations within fermentation cellars were collected, and the yeast communities therein were assessed via culture-based and denaturing gradient gel electrophoresis (DGGE) approaches. These analyses revealed significant differences in the composition of yeast communities present in different layers of pit mud. In total, 29 different yeast species were detected, and principal component analyses revealed clear differences in microbial diversity in pit mud samples taken from different cellar locations. Culture-dependent strategies similarly detected 20 different yeast species in these samples. However, while *Geotrichum silvicola*, *Torulaspora delbrueckii*, *Hanseniaspora uvarum*, *Saturnispora silvae*, *Issatchenkia orientalis*, *Candida mucifera*, *Kazachstania barnettii*, *Cyberlindnera jadinii*, *Hanseniaspora* spp., *Alternaria tenuissima, Cryptococcus laurentii*, *Metschnikowia spp*., and *Rhodotorula dairenensis* were detected via a PCR-DGGE approach, they were not detectable in culture-dependent analyses. In contrast, culture-based approaches led to the identification of *Schizosaccharomyces pombe* and *Debaryomyces hansenii* in these pit mud samples, whereas they were not detected using DGGE fingerprints profiles. An additional HS-SPME-GC-MS-based analysis of the volatile compounds present in fermented grains samples led to the identification of 66 such compounds, with the highest levels of volatile acids, esters, and alcohols being detected in fermented grains from lower layer samples. A canonical correspondence analysis (CCA) suggested they were significant correlations between pit mud yeast communities and associated volatile compounds in fermented grains.

**Supplementary Information:**

The online version contains supplementary material available at 10.1186/s13568-023-01562-7.

## Introduction

Chinese liquor is a traditional fermented distilled spirit that is widely consumed in China and plays an important role in Chinese culture. Owing to its unique flavor profile, it is also becoming increasingly popular in other areas in East Asia. The flavor characteristics of different liquor preparations allow them to be classified into 12 different categories, including soy sauce flavour, strong flavour, light flavour, and miscellaneous flavour types (Xu et al. 2017). Of these, Chinese strong-flavour liquor is the most popular owing to its strong aroma and sweet flavour, accounting for roughly 70% of total liquor consumption in China (Pu and Yan 2022). Chinese strong-flavour liquor is produced via the distillation of a mixture of fermented grains including what, sorghum, and rice in a specialized fermentation pit (about 3.4 m long, 1.8 m wide, and 2.0 m deep) containing bacteria, archaea, and fungi. The walls and bottom of this fermentation pit are covered with pit mud, which is a type of fermented clay containing an array of anaerobic microbes. During the fermentation process, this pit mud supports the growth of microbes responsible for generating the volatile compounds that give Chinese strong-flavour liquor its unique taste (Tao et al. 2017). The composition of pit mud microbial communities thus determines the quality and flavour of the resultant liquor. Individual fermentation cellars are generally used for many years, and the fermented grains placed in the lower portion of the cellar can help to produce high-quality Chinese strong-flavour liquor. Prior studies have shown that microbial diversity is significantly increased in pit mud samples from the bottom of these fermentation cellars relative to samples from the upper wall pit layer (Ding et al. 2016). It is generally understood that the best Chinese strong-flavour liquor is also generated in the lower portion of the pit closer to the fermented grains, emphasizing the importance of the composition of pit mud along the lower walls and bottom of the cellar on Chinese strong-flavour liquor fermentation. Location-dependent effects on the production of Chinese strong-flavour liquor are thought to be attributable to the microbial domestication that occurs within a given fermentation pit during the process of recycling fermentation (Zhang et al. 2017), therefore, it is necessary to clarify the mechanisms underlying these effects and to investigate pit mud microbial composition.

Both culture-dependent and -independent strategies have previously been employed to study pit mud microbial communities. An early culture-based study identified *Clostridium sp*. W1 as the primary caproic acid-producing bacteria presented in Wuliangye liquor pit mud (Xue et al. 1988), while pit mud samples associated with the production of Luzhou Laojia liquor were dominated by by *Hydrogenispora* (57.2%), *Sedimentibacter* (5.4%), and *Caproiciproducens* (4.9%) (Qian et al. 2020). A range of bacteria, fungi, and archaea have been detected in pit mud samples (Xiao et al. 2023). In an effort to better understand time-dependent changes in these pit mud microbial communities, Tao et al. (2014) studied pit mud samples from pits that were 1, 10, 25, and 50 years old, revealing an upward trend in microbial diversity with pit age that plateaued after 25 years. Zhang et al. (2020) similarly conducted a multidimensional analysis of microbial communities in older and younger pit mud samples, and found that microbial diversity varied significantly as a function of vertical depth but not horizontal position within a given pit. Specifically, they found pit mud samples from the center of the pit were dominated by Lactobacillus species (12.80-42.72%), whereas those from the corner were dominated by caproiciproducens species (17.85-64.45%). These researchers ultimately determined that the factors most important for regulating pit mud microbial growth were pH, lactic acid, and soluble Ca^2+^ concentrations. Zhang et al. (2015) utilized culture-independent strategies including nested PCR-denaturing gradient gel electrophoresis (PCR-DGGE), phospholipid fatty acid (PLFA), phospholipid ether lipids (PLEL), and fluorescence in situ hybridization (FISH) analyses to characterize microbial communities in samples of artificial pit mud (APM) used to brew Chinese strong-flavour liquor. dominant bacteria in these samples included *Clostridiales*, *Lactobacillales*, *Bacteroidales*, and *Rhizobiales* species, while archaea present therein included *Methanomicrobiales* and *Methanosarcinales* species, and fungi included *Saccharomycetales* and *Eurotiales* species. They additionally determined that the pattern of APM piling influenced the consequent microbial community structure in a given sample. While many prior studies have explored bacterial community structures and functional properties in pit mud samples, there have been fewer analyses to date of pit mud yeast communities or the impact of cellar spatial locations on these community structures.

Yeast plays an important role in the preparation of Chinese liquor, controlling both the fermentation rate and the flavour profile of the resultant brew through the metabolic processing of different nutrients into volatile compounds (Wang et al. 2019). However, pit mud yeast diversity in the context of strong-flavour liquor production is poorly understood, as are the yeast-derived volatile compounds that ultimately contribute to liquor flavour.

In the present study, we employed a PCR-DGGE approach to study the structures of yeast communities in pit mud samples from different fermentation cellar depths. In addition, a head space-solid phase micro-extraction combined gas chromatography-mass spectrometry (HS-SPME-GC-MS) approach was additionally used to identify volatile compounds in liquor samples from upper, middle, and lower layers of fermented grains fermentation. Correlations between identified yeast communities and liquor flavour compounds were additionally assessed. Overall, the results of this study will offer new insights regarding the role of pit mud yeast communities in Chinese strong-flavour liquor production.

## Materials and methods

### Samples of pit mud and fermented grains

Pit mud samples were collected from a famous Chinese strong-flavour liquor distilleries located in Anhui provinces, China, and the pit ages was about 20 years. Samples were taken from the wall or bottom of the pits. The source, cellar age and sampling location of the pit muds are shown in Fig. S1. Each sample plot was divided into eight subplots (centre and edges) except bottom with nine subplots (side centre, side edges and bottom middle), and about 100 g of pit mud was collected from each subplot, then eight or nine subsamples were sufficiently mixed. The sampling depth of each subplot was about 5 cm.

Additionally, the fermented grains samples were taken respectively from the center of the top, middle and bottom layer of the fermentation pit filled with multiple-grains at the end of the fermentation. Finally, all samples were transferred to sterile polyethylene bags without air, sealed and stored at -20 °C until used.

### Examination of yeast community

#### DNA extraction

Extraction total DNA from pit mud was performed by modified methods of Tan et al. (2020). Briefly, pit mud (5 g) was mixed with 15 mL CTAB solution and 100 µL protease K (10 mg/mL) and shaken horizontally at 225 rpm at 30 °C for 30 min. After the shaking, 1.5 mL 20% SDS was added and the mixture was incubated at 65 °C for 120 min, and then was inverted gently every 15 min. After centrifugation at 8,000 × g for 5 min at room temperature, the supernatant was mixed with an equal volume of chloroform/isoamyl/alcoholsolution (25: 24: 1). The mixture was centrifuged at 8,000 × g at room temperature for 5 min. Isopropanol (0.6-1.0× supernatant volume) and the mixture were incubated for 60 min at room temperature. Precipitates were collected by centrifugation at 20,000 x g for 20 min at room temperature, washed twice with 70% (v/v) ethanol and resuspended in sterile deionised water to a final volume of 200 µL. The DNA was purified using Universal UNlQ-10 Column DNA Purification Kit (Sangon, Shanghai, China) and quantified using a NanoDrop spectrophotometer (Thermo Fisher Scientific, Carlsbad, CA, USA).

### PCR amplification

For yeast diversity analysis, the D1/D2 domain of the 26 S rRNA gene was amplified using universal primers NL1 (5′-GCGATATCAATAAGCGGAGGAAAAG-3′) and NL4 (5′-GGTCCGTGTTTCAAGACGG-3′) in the first round of the nested PCR approach according to Yan et al. (2019). Subsequently, this initial PCR product was diluted and used as a template for a nested PCR with primers NL1 containing a GC-clamp (5′-CGCCCGGGGCGCGCCCCGGGGCGGGGCGGGGGCGCGGGGGG-3′) at the 5′ end and LS-2 (5′-ATTCCCAAACAACTCGACTC-3′) (Nielsen et al. 2007). All reactions were carried out in a 50 µL volume containing 5 µL 10× PCR reaction buffer, 3.2 µL dNTP Mixture (2.5 mM), 0.4 µL ExTaq (5 U/µL), 50 ng DNA template, 1 µL of each primer (20 µM), and double deionized wate for adjustment of the volume to 50 µL. The first PCR amplification conditions was performed as follows: initial denaturation at 94 °C for 3 min, then 35 cycles of denaturation at 94 °C for 35 s, annealing at 50 °C for 35 s, extension at 72 °C for 1 min and 10 s; extension at 70 °C for 10 min. The second PCR amplification conditions was the same with the first PCR process except that the conditions of annealing at 60 − 55 °C for 35 s. The PCR products were then purified using a SanPrep Column PCR Product Purification Kit (Sangon, Shanghai, China). Before applied to DGGE analysis, all the PCR products were examined by electrophoresis on 1% agarose gels with ethidium bromide.

### DGGE analysis

DGGE analysis of the PCR products was performed on a DCode Universal Mutation Detection System (Bio-Rad, Hercules, CA, USA). Polyacrylamide gels (7% w/v acrylamide–bisarylamide) were prepared with a Bio-Rad Gradient Delivery System (Model 475, Bio-Rad) using solutions containing 40% and 60% denaturant (100% denaturant corresponds to 7 M urea and 40% v/v formamide). Gels were run at 60 °C for 5 h at 150 V. The amplified fragments were visualized by AgNO_3_ solution staining and UV transillumination (Yan et al. 2019). The yeast fingerprint on the DGGE gel was analyzed using the Quantity one software (Bio-Rad).

### Excision of DGGE bands and sequencing

The predominant DGGE bands observed in the DGGE profiles were excised and eluted in ultrapure water at 4 °C overnight, and the eluted DNA was re-amplified using the second round primers mentioned in 2.2.2 without GC clamp. The PCR products were purified with a universal PCR purification kit (Tiangen, Beijing, China) Then the purified DNA was ligated into a pGEM-T easy vector and transformed into competent Escherichia coli DH5a cells according to the manufacturer’s instructions and the laboratory manual. Inserts from white colonies were amplified by adding whole cells directly to PCR reactions using the primer set M13F and M13 R (Sangon, Shanghai, China) as described by Liu et al. (2012). All positive colonies extracted from white colonies were sequenced by an automated DNA sequencer (Sangon, Shanghai, China). Subsequently, GenBank BLAST (http://www.ncbi.nlm.nih.gov/BLAST) was performed to identify the closest phylogenetic relatives of the partial rDNA sequences tested above.

### Data analysis

The DGGE bands intensity and similarity matrix of DGGE profiles were calculated and exported out using Quantity one software (Bio-Rad). The community diversity indices including Shannon–Wiener index of general diversity (H), the Evenness (E), and the species richness (S) were calculated according to previous protocols (Yan et al. 2019). The dendrograms were calculated on the basis of Dice’s coefficient of similarity (weighted data), using the unweighted pair group method with arithmetic averages clustering algorithm (UPGMA).

### Enumeration and isolation of yeasts

Yeasts were isolated and quantified using spread plates. Ten grams of pit mud sample were homogenized with 90mL sterile distilled water and the mixture was incubated at 25 °C for 30 min with shaking at 180 rpm. Diluted suspension (100 µL) was plated on YPD agar (10 g/L yeast extract, 20 g/L peptone, 20 g/L glucose and 20 g/L agar) supplemented with 100 µ g/mL ampicillin for yeasts. All assays were in triplicate. The yeasts were incubated at 30 °C for 2 days. Colonies were identified by their morphology and by performing PCR with primer pairs ITS1/ITS4 (ITS1: TCCGTAGGTGAACCTGCGG, ITS4: TCCTCCGCTTATTGATATGC) for yeast (Li et al. 2021). Sequence identity was analyzed with a GenBank search (http://www.ncbi.nlm.nih.gov/BLAST/).

### HS-SPME-GC-MS analysis of fermented grains

The liquor samples, respectively collected from the distillation of the up, middle, and bottom layer of fermented grains, were detected via headspace solid-phase micro-extraction (HS-SPME) combined with gas chromatography mass spectrometry (GC-MS). HS-SPME was performed under previously reported conditions with slight modifications (Yan et al. 2019). A 5.0mL liquor sample diluted to 10% ethanol by volume, was transferred to a 20.0mL conical bottomed glass vial, then saturated with NaCl (1.5 g). After 100µL 2-octanol (70 mg/L, internal standard) solution was was injected into the the vial, the mixture were equilibrated by ultrasonic vibration in 50 °C constant temperature water bath for 10 min. After that, the extraction head was then inserted into each vial, and the sample was extracted at 60 °C for 30 min.

After HS-SPME, the extraction head was inserted into the injection port of the GC-MS system (Agilent 6890 GC and Agilent 5975 mass selective detector (MSD); Agilent, San Diego, USA) to separate and analyze the different compounds in the extracts. GC-MS was performed as previously reported with slight modifications (Yan et al. 2020). The samples were separated through a DB-Wax column (60 m length, 0.25 mm internal diameter, 0.25 μm film thickness) using helium as the carrier gas at a constant flow rate of 1 mL/min. The column temperature was programmed as follows: 40 °C for 2 min, increased by 5 °C/min to 80 °C for 2 min, and again increased by 8 °C/min to 230 °C for 7 min. High-purity nitrogen was applied as eluant gas to split sampling with a split ratio of 30: 1. The ionization energy was set equal to 70 eV, and the ion source and quadruple temperatures were set at 200 and 250 °C respectively. MS spectra were performed in scan mode (33–450 amu). Each sample was analyzed in triplicate.

## Results

### DGGE-based yeast community detection

To gain comprehensive insights regarding yeast spatial distributions, we next analyzed yeast community structures in pit mud samples from the upper, middle, lower, and bottom cellar layers via a PCR-DGGE approach which enabled us to calculate yeast diversity indices associated with these different spatial distributions (Table 1). We found that species richness was highest for samples from the upper pit mud layer, followed by that of samples from the bottom layer. Samples from the upper and bottom laters also exhibited higher levels of evenness relative to samples from the middle and lower levels (Table 1). Samples from the upper and bottom pit mud layers also had higher Shannon–Wiener index values than middle and lower layer samples, with samples from the Shannon-Wiener index value (3.03).

**Table 1:** Indices of yeast community in the samples collected from different spatial positions of cellar according to quantified bands from Fig. 1

Lane ^a^	Shannon-Wiener	Evenness	Richness
U	3.03	0.98	19
M	2.77	0.94	17
D	2.33	0.94	8
B	2.97	0.98	17

^a^ Lanes U, M, D, and B respectively represent pit mud samples collected from up wall layer of cellar, middle wall layer of cellar, down wall layer of cellar, and bottom layer of cellar, and were sampled from the same fermentation cellar

**Fig. 1:**
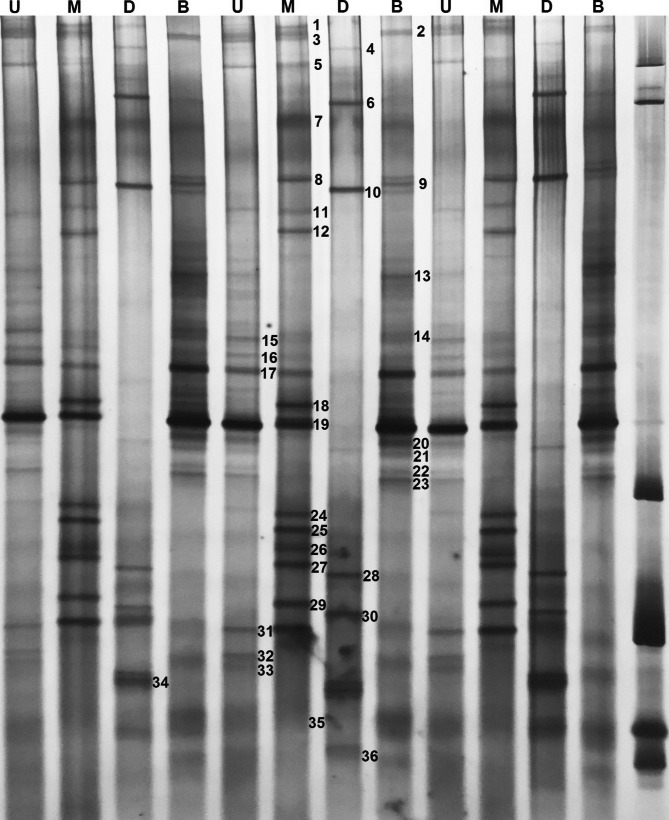
Denaturing gradient gel electrophoresis (DGGE) pattern of yeast 26 S rRNA in the pit mud samples collected from different spatial positions of cellar. Lanes U, M, D, and B represent samples collected from up wall layer of cellar, middle wall layer of cellar, down wall layer of cellar, and bottom layer of cellar, respectively. The bands indicated with numbers were excised and sequenced and the alignment results are listed in Table 2

**Table 2:** Identities of 26 S rRNA sequences of DGGE bands *via* BLAST

Band no.^a^	Closest relative (NCBI accession no.)	Identity (%)^b^
1	*Geotrichum silvicola* (NG_060622.1)	99.0
2	*Geotrichum silvicola* (MW233050.1)	99.0
3	*Geotrichum silvicola* (MW233034.1)	99.0
4	*Torulaspora delbrueckii* (MH010872.1)	98.5
5	*Issatchenkia orientalis* (DM138225.1)	99.0
6	*Hanseniaspora uvarum* (MT707264.1)	99.6
7	*Saturnispora silvae* (EF550215.1)	98.5
8	*Geotrichum bryndzae* (EU429455.1)	99.0
9	*Geotrichum bryndzae* (LC171719.1)	98.3
10	*Saccharomycopsis fibuligera* (LY516482.1)	98.0
11	*Pichia anomala* (AY349451.1)	99.1
12	*Pichia farinosa* (FN555626.1)	99.0
13	*Issatchenkia orientalis* (KX131152.1)	98.0
14	*Alternaria tenuissima* (MF405157.1)	98.5
15	*Candida mucifera* (AB041006.1)	98.2
16	*Yarrowia lipolytica* (AL411863.1)	99.3
17	*Wickerhamomyces anomalus* (HG316786.1)	99.4
18	*Candida intermedia* (MW165041.1)	99.4
19	*Pichia kudriavzevii* (KX023220.1)	99.0
20	*Pichia kudriavzevii* (KX015902.1)	99.0
21	*Pichia occidentalis* (EF550236.1)	100.0
22	*Trichosporon asahii* (KR872659.1)	99.3
23	*Trichosporon asahii* (KR872657.1)	99.1
24	*Kazachstania barnettii* (MW477711.1)	99.0
25	*Pichia guilliermondii* (AF218967.1)	99.3
26	*Hanseniaspora spp.* (MH681740.1)	99.5
27	*Candida humilis* (AF402039.1)	98.0
28	*Candida tropicalis* (LX265350.1)	99.2
29	*Cyberlindnera jadinii* (KX015911.1)	98.0
30	*Hanseniaspora vineae* (LC474406.1)	99.0
31	*Cryptococcus laurentii* (JX394003.1)	98.5
32	*Cryptococcus laurentii* (JX394000.1)	98.0
33	*Metschnikowia spp.* (AY313961.1)	98.3
34	*Rhodotorula dairenensis* (MW487320.1)	99.5
35	*Saccharomyces cerevisiae* (AF458979.1)	99.1
36	*Saccharomyces cerevisiae* (AF458976.1)	98.3

^a^ Band(s) are numbered as indicated on the DGGE fingerprint files shown in Fig. 1; ^b^ Accession number of the sequence of the closet relative found in NCBI database

**Fig. 2:**
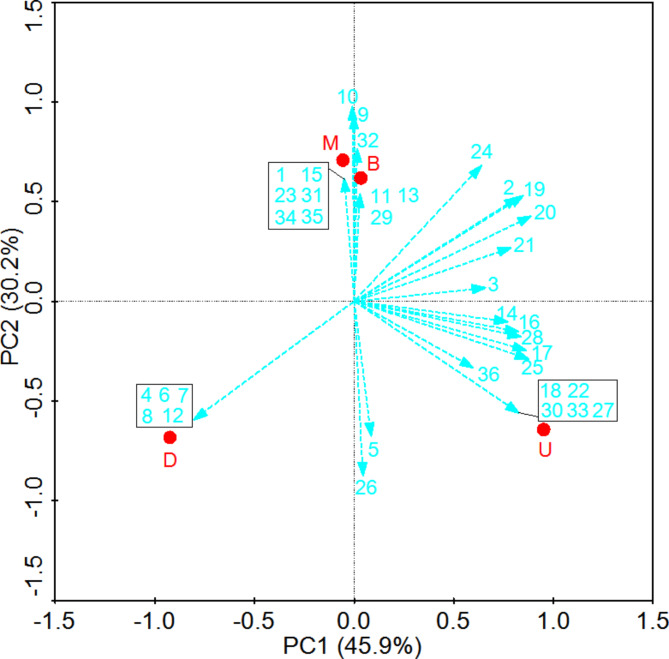
Principal component analysis of yeast communities on three layers of pit mud samples. The first principal component (X axis) explains 45.9% of the total variance of the dataset, while the second principal component (Y axis) explains 30.2% of the total variance of the dataset. Yeasts are numbered as indicated on the DGGE fingerprint files shown in Fig. 2; Table 2; U, M, D, and B, represent up, middle, down, and bottom layer of pit mud, respectively

In total, 36 dominant bands were identified in DGGE profiles (labeled from 1 to 36 in Fig. 2). These bands were then sequenced and compared to the GenBank database (Table 2, supplementary materials 2). This revealed that the upper pit mud samples contained high levels of *Saturnispora silvae* (band 7), *Geotrichum bryndzae* (band 8), *Pichia farinosa* (bands 12), *Candida intermedia* (band 18), *Pichia kudriavzevii* (band 19), *Kazachstania barnettii* (band 24), *Pichia guilliermondii* (band 25), *Hanseniaspora spp.* (band 26), *Candida humilis* (band 27), *Cyberlindnera jadinii* (band 29), and *Cryptococcus laurentii* (band 31), whereas they were present at low levels or were absent in other layers. In the middle layer of pit mud, *Hanseniaspora uvarum* (band 6), *Saccharomycopsis fibuligera* (band 10), *Candida tropicalis* (band 28), *Hanseniaspora vineae* (band 30), and *Rhodotorula dairenensis* (band 34) were present at higher layers than in other samples with the exception of *Pichia kudriavzevii* (band 19). In lower layer samples, *Wickerhamomyces anomalus* (band 17), *Pichia kudriavzevii* (band 19), and *Pichia kudriavzevii* (band 20) were dominant, with *Pichia kudriavzevii* (band 19) being present at higher levels than in other samples. *Pichia kudriavzevii* (band 19) were also present at high levels in bottom layer pit mud samples. As *Pichia kudriavzevii* (band 19) was present in all samples other than the middle layer, suggesting they may be a key member of the yeast pit mud flora.

We next performed a PCA analysis of the data in Fig. 2, revealing clear microbial community-dependent discrimination between pit mud samples from different physical locations within the fermentation cellar (Fig. 1). Yeast composition profiles separated these pit mud samples into these three groups, each exhibiting unique microbial diversity.

In total, 20 yeast species were detected in pit mud via our culture-dependent approach (Table 3). However, certain species (*Geotrichum silvicola*, *Torulaspora delbrueckii, Hanseniaspora uvarum*, *Saturnispora silvae, Issatchenkia orientalis*, *Candida mucifera, Kazachstania barnettii*, *Cyberlindnera jadinii, Hanseniaspora spp. Alternaria tenuissima, Cryptococcus laurentii*, *Metschnikowia spp.*, and *Rhodotorula dairenensis*) that we detected in our initial DGGE analysis were not isolated via the present culture-bassed method. This may suggest that the utilized culture medium was not appropriate for these yeast species, or that they were no longer viable in analyzed samples. Future studies of culture media selectivity will be necessary to more fully understand pit mud microecology.

**Table 3:** Isolated yeast strains identities following purification

No.	GenBank accession number	Sequence similarity (%)	Closest relative
YE001	MW076944	100	*Hanseniaspora vineae*
YE002	MW076945	99	*Pichia kluyveri*
YE003	MW076946	100	*Trichosporon asahii*
YE004	MW076947	100	*Pichia kluyveri*
YE005	MW076948	100	*Hanseniaspora vineae*
YE006	MW076949	100	*Saccharomyces cerevisiae*
YE007	MW076950	100	*Wickerhamomyces anomalus*
YE008	MW076951	100	*Kluyveromyces lactis*
YE009	MW076952	100	*Saccharomyces cerevisiae*
YE010	MW076953	100	*Wickerhamomyces anomalus*
YE011	MW076954	100	*Yarrowia lipolytica*
YE012	MW076955	100	*Wickerhamomyces mori*
YE013	MW076956	100	*Galactomyces geotrichum*
YE014	MW076957	100	*Dabaryomyces hansenii*
YE015	MW076958	100	*Wickerhamomyces mori*
YE016	MW076959	100	*Saccharomyces kudriavzevii*

We additionally noted that certain species detected via our culture-dependent approach (*Schizosaccharomyces pombe* and *Debaryomyces hansenii*) were not evident in the above DGGE fingerprints profiles. This may be a consequence of differences in sample handling protocols that impacted microbial growth or viability, such as variations in sample temperature or aerobic/anaerobic storage (Zhang et al. 2016). The PCR-DGG approach also has a detection limit of 10^4^-10^8^ cfu/mL (Ercolini 2004). As such, microbe concentrations and numbers and pit mud may limit our ability to detect less abundant species via DGGE as a consequence of changes in DNA extraction and PCR amplification efficiency.

Many of the yeast species identified in the present analysis were also detected in our prior analysis of the microbial communities in *Daqu*-starter samples (Yan et al. 2019). *Daqu*-starter contains large quantities of yeast, making it a valuable crude microorganism source accounting for 10–20% of the raw material used in liquor production. We therefore speculate that pit mud microbial communities are derived in large part from the initial *Daqu*-starter.

### Assessment of spatial volatile compound profiles in fermented grains samples

In total, 66 different volatile compounds were detected via HS-SPME-GC-MS in analyzed samples collected from the upper, middle, and bottom layers of fermented grains, including 14 acids, 19 esters, 18 alcohols, 6 aldehydes, 2 ketones, 5 alkanes, and 2 volatile phenols (Table 4).

**Table 4:** The volatile aroma compounds detected and measured in the samples collected from different spatial positions of fermented grain

Number	Aroma compounds	Retention time (min)	Identification	Contents of volatile aroma compounds of fermented grain /(µg/mg)		
				UZ	MZ	DZ
	*Volatile acids*					
AC1	Acetic acid	9.879	MS, RI	2.535 ± 0.125a	5.287 ± 0.258b	8.387 ± 0.312c
AC2	Propionic acid	12.377	MS, RI	0.765 ± 0.114a	2.154 ± 0.127b	4.154 ± 0.205c
AC3	Butyric acid	14.913	MS, RI	1.163 ± 0.054a	2.854 ± 0.241b	4.676 ± 0.302c
AC4	Caproic acid	16.214	MS, RI	1.167 ± 0.126a	3.951 ± 0.235b	6.765 ± 0.478c
AC5	3-methyl-pentanoic acid	16.389	MS, RI	0.000a	0.625 ± 0.068b	0.958 ± 0.056c
AC6	2-methyl-butanoic acid	15.588	MS, RI	0.000a	0.487 ± 0.084b	1.254 ± 0.214c
AC7	Octanol acid	19.512	MS, RI	0.120 ± 0.015a	0.127 ± 0.016a	0.234 ± 0.024b
AC8	2-Methyl butanoic acid	24.102	MS, RI	0.102 ± 0.018a	0.312 ± 0.028b	0.425 ± 0.036c
AC9	Pentanoic acid	25.278	MS, RI	0.212 ± 0.019a	0.247 ± 0.021a	0.257 ± 0.026a
AC10	Nonanoic acid	26.761	MS, RI	0.117 ± 0.019a	0.215 ± 0.026b	0.250 ± 0.035b
AC11	Hexanoic acid	27.37	MS, RI	0.112 ± 0.010a	0.117 ± 0.013a	0.225 ± 0.012b
AC12	Palmitic acid	34.615	MS, RI	0.323 ± 0.056a	0.368 ± 0.038a	0.389 ± 0.040a
AC13	Octanoic acid	35.021	MS, RI	0.035 ± 0.008a	0.126 ± 0.016a	0.225 ± 0.201a
AC14	Decanoic acid	35.41	MS, RI	0.087 ± 0.005a	0.158 ± 0.021b	0.299 ± 0.015c
	Σ			6.738 ± 0.102	17.028 ± 0.189	28.498 ± 0.313
	*Esters*					
ES1	Ethyl acetate	4.032	MS, RI	6.465 ± 0.987a	10.325 ± 1.023b	16.421 ± 1.213
ES2	Ethyl isobutanoat	5.567	MS, RI	0.287 ± 0.014a	1.743 ± 0.214b	0.712 ± 0.052c
ES3	Ethyl butanoate	5.443	MS, RI	0.353 ± 0.068a	1.557 ± 0.168b	2.832 ± 0.254c
ES4	Ethyl hexanoate	6.049	MS, RI	4.725 ± 0.365a	8.876 ± 1.021b	17.154 ± 1.232c
ES5	Ethyl oenanthate	8.239	MS, RI	0.435 ± 0.032a	0.792 ± 0.058b	1.526 ± 0.140c
ES6	Ethyl 2-methylbutanoate	10.285	MS, RI	0.526 ± 0.023a	1.158 ± 0.101b	1.988 ± 0.187c
ES7	Ethyl 3-methylbutanoate	10.602	MS, RI	0.468 ± 0.036a	1.025 ± 0.124b	1.854 ± 0.112c
ES8	Nonanoic acid ethyl ester	12.976	MS, RI	0.821 ± 0.054a	1.287 ± 0.068b	2.321 ± 0.096c
ES9	Ethyl decanoate	15.317	MS, RI	1.053 ± 0.036a	1.087 ± 0.057a	0.993 ± 0.065a
ES10	Ethyl heptanoate	16.862	MS, RI	1.024 ± 0.152a	2.012 ± 0.185b	3.214 ± 0.220c
ES11	Benzeneacetic acid ethyl ester	18.623	MS, RI	1.587 ± 0.702a	1.256 ± 0.075a	1.032 ± 0.065a
ES12	Ethyl laurate	19.752	MS, RI	1.021 ± 0.098a	1.512 ± 0.103b	1.997 ± 0.121c
ES13	γ-nonylactone	23.442	MS, RI	0.432 ± 0.036a	0.556 ± 0.045b	0.952 ± 0.051c
ES14	Ethyl oleate	23.726	MS, RI	4.337 ± 0.401a	4.258 ± 0.398a	4.361 ± 0.385a
ES15	Ethyl pentadecanoate	25.764	MS, RI	3.668 ± 0.258a	3.174 ± 0.261b	2.189 ± 0.187c
ES16	Ethyl 9-hexadecenoate	28.161	MS, RI	3.327 ± 0.257a	3.418 ± 0.264a	3.032 ± 0.213a
ES17	Ethyl palmitate	28.268	MS, RI	5.698 ± 0.445a	5.735 ± 0.406a	5.676 ± 0.412a
ES18	Ethyl linoleate	31.429	MS, RI	4.977 ± 0.235a	4.985 ± 0.236a	5.034 ± 0.239a
ES19	Ethyl octadecanoate	38.895	MS, RI	1.254 ± 0.132a	2.145 ± 0.201b	3.210 ± 0.254c
	Σ			42.458 ± 0.875	56.901 ± 0.497	76.498 ± 0.687
	*Alcohols*					
AL1	3-methyl-butanol	5.583	MS, RI	0.352 ± 0.065a	0.732 ± 0.045b	1.597 ± 0.036c
AL2	Isoamyl alcohol	5.842	MS, RI	1.523 ± 0.116a	0.736 ± 0.085b	0.474 ± 0.036c
AL3	1-hexanol	7.281	MS, RI	0.226 ± 0.036a	0.276 ± 0.028ab	0.197 ± 0.027b
AL4	2-methyl-1-propanol	10.321	MS, RI	0.215 ± 0.036a	0.621 ± 0.045b	0.889 ± 0.061c
AL5	1-octen-3-ol	11.11	MS, RI	0.688 ± 0.052a	0.379 ± 0.029b	0.223 ± 0.028c
AL6	Isobutanol	11.34	MS, RI	0.215 ± 0.018a	0.356 ± 0.031b	0.625 ± 0.048c
AL7	Enanthol	11.223	MS, RI	0.263 ± 0.021a	0.255 ± 0.019a	0.275 ± 0.018a
AL8	Isooctanol	12.107	MS, RI	0.389 ± 0.028a	0.279 ± 0.019b	0.201 ± 0.017c
AL9	1-Butanol	12.78	MS, RI	0.158 ± 0.015a	0.268 ± 0.021b	0.441 ± 0.034c
AL10	2,3-butanediol	13.399	MS, RI	0.000a	1.525 ± 0.102b	3.085 ± 0.231c
AL11	Octanol	13.611	MS, RI	0.378 ± 0.027a	0.204 ± 0.019b	0.125 ± 0.014c
AL12	2-Methylbutanol	14.11	MS, RI	0.000a	1.131 ± 0.132b	2.231 ± 0.242c
AL13	Isopentanol	14.15	MS, RI	1.257 ± 0.015a	0.654 ± 0.026b	0.364 ± 0.017c
AL14	1-Pentanol	15.09	MS, RI	0.357 ± 0.028a	0.674 ± 0.045b	1.025 ± 0.103c
AL15	1-nonanol	15.966	MS, RI	0.232 ± 0.031a	0.167 ± 0.013a	0.154 ± 0.018b
AL16	2-Heptanol	16.60	MS, RI	0.126 ± 0.023a	0.265 ± 0.019b	0.398 ± 0.025c
AL17	Benzyl alcohol	20.635	MS, RI	0.929 ± 0.058a	0.631 ± 0.054b	0.356 ± 0.026c
AL18	Phenylethyl alcohol	21.304	MS, RI	0.000a	2.351 ± 0.215b	5.705 ± 0.498c
	Σ			7.308 ± 0.081	11.504 ± 0.098	18.365 ± 0.254
	*Aldehydes*					
AD1	2-Heptenal	7.884	MS, RI	0.315 ± 0.029a	0.267 ± 0.025b	0.132 ± 0.012c
AD2	Nonaldehyde	9.446	MS, RI	0.164 ± 0.015a	0.187 ± 0.013b	0.116 ± 0.012b
AD3	Benzaldehyde	12.712	MS, RI	1.267 ± 0.015a	2.336 ± 0.035b	0.363 ± 0.021c
AD4	2-undecenal	17.689	MS, RI	0.278 ± 0.027a	0.857 ± 0.068b	0.101 ± 0.011c
AD5	Pentanal	18.148	MS, RI	0.317 ± 0.026a	0.399 ± 0.038a	0.267 ± 0.019b
AD6	2-phenyl-2-butenal	21.577	MS, RI	1.632 ± 0.032a	1.276 ± 0.017b	2.223 ± 0.021c
	Σ			3.973 ± 0.025	5.322 ± 0.031	3.202 ± 0.014
	*Ketones*					
KE1	2-octanone	6.963	MS, RI	1.903 ± 0.112a	2.231 ± 0.118b	1.412 ± 0.116c
KE2	2-nonanone	9.354	MS, RI	0.182 ± 0.017a	0.262 ± 0.026a	0.179 ± 0.016b
	Σ			2.085 ± 0.562	2.493 ± 0.601	1.591 ± 0.573
	*Alkanes*					
AK1	Decamethylcyclopentasiloxane	4.782	MS, RI	0.215 ± 0.021a	0.826 ± 0.045b	0.616 ± 0.057c
AK2	Tetradecane	9.660	MS, RI	0.513 ± 0.046a	0.757 ± 0.037a	0.512 ± 0.045b
AK3	Pentadecane	12.018	MS, RI	1.761 ± 0.116a	1.451 ± 0.116b	1.782 ± 0.124b
AK4	Caryophyllene	14.097	MS, RI	1.021 ± 0.012a	2.357 ± 0.023b	0.669 ± 0.063c
AK5	Hexadecane	14.332	MS, RI	0.587 ± 0.038a	1.383 ± 0.054b	0.798 ± 0.062c
	Σ			4.097 ± 0.054	6.774 ± 0.041	4.377 ± 0.058
	*Volatile phenols*					
VP1	Phenol	24.562	MS, RI	0.185 ± 0.019a	0.875 ± 0.013b	0.231 ± 0.014c
VP2	2-Methoxy-4-vinylphenol	26.754	MS, RI	0.000a	0.275 ± 0.035b	0.426 ± 0.062c
	Σ			0.185 ± 0.019	1.15 ± 0.029	0.657 ± 0.054

Note: UZ, MZ, and DZ, represent the samples collected from up, middle, and down layer of fermented grain, respectively. The data were presented as mean ± standard deviations, different small letters in the same column represent significant differences at 0.05 level

Of the 14 acids detected in the middle and bottom fermented grains layers, the levels of acetic acid were highest in all three layers, while 2-methyl-butanoic acid and 3-methyl-pentanoic acid were present only in the middle and bottom layers and not in the upper layer.

Esters were the most abundant and important aroma compounds in these fermented grains samples. We found that levels of ethyl acetate, ethyl hexanoate, ethyl butanoate, ethyl hexanoate, ethyl oenanthate, ethyl 2-methylbutanoate, ethyl 3-methylbutanoate, nonanoic acid ethyl ester, ethyl heptanoate, ethyl laurate, γ-nonylactone, and ethyl octadecanoate were highest in samples collected from the bottom layer of fermented grains, followed by levels the middle layer. Ethyl isobutanoat levels were highest in the middle layer of fermented grains, while benzeneacetic acid ethyl ester and ethyl pentadecanoate were present at the highest levels in the upper layer. Levels of ethyl decanoate, ethyl oleate, ethyl 9-hexadecenoate, ethyl palmitate, and ethyl linoleate did not differ significantly among fermented grains layers.

Alcohols were also present at high levels in fermented grains samples, as shown in Table 4. Levels of 3-methyl-butanol, 2-methyl-1-propanol, isobutanol, 1-butanol, 2,3-butanediol, 2-methylbutanol, 1-pentanol, 2-methylbutanol, 1-pentanol, 2-heptanol, and phenylethyl alcohol in the bottom fermented grains layer were significantly higher than those in other layers, while the middle layer contained the highest levels of 1-hexanol, and the upper layer contained the highest levels of isoamyl alcohol, 1-octen-3-ol, isooctanol, octanol, isopentanol, 1-nonanol, and benzyl alcohol. Ethanol levels did not differ significantly among fermented grains layers.

The highest total levels of other volatile compounds such as aldehydes, ketones, alkanes, and volatile phenols were detected in the middle layer of fermented grains, with the second highest levels being detected in the bottom fermented grains layer, whereas these levels were lowest in the upper fermented grains layer.

**Fig. 3:**
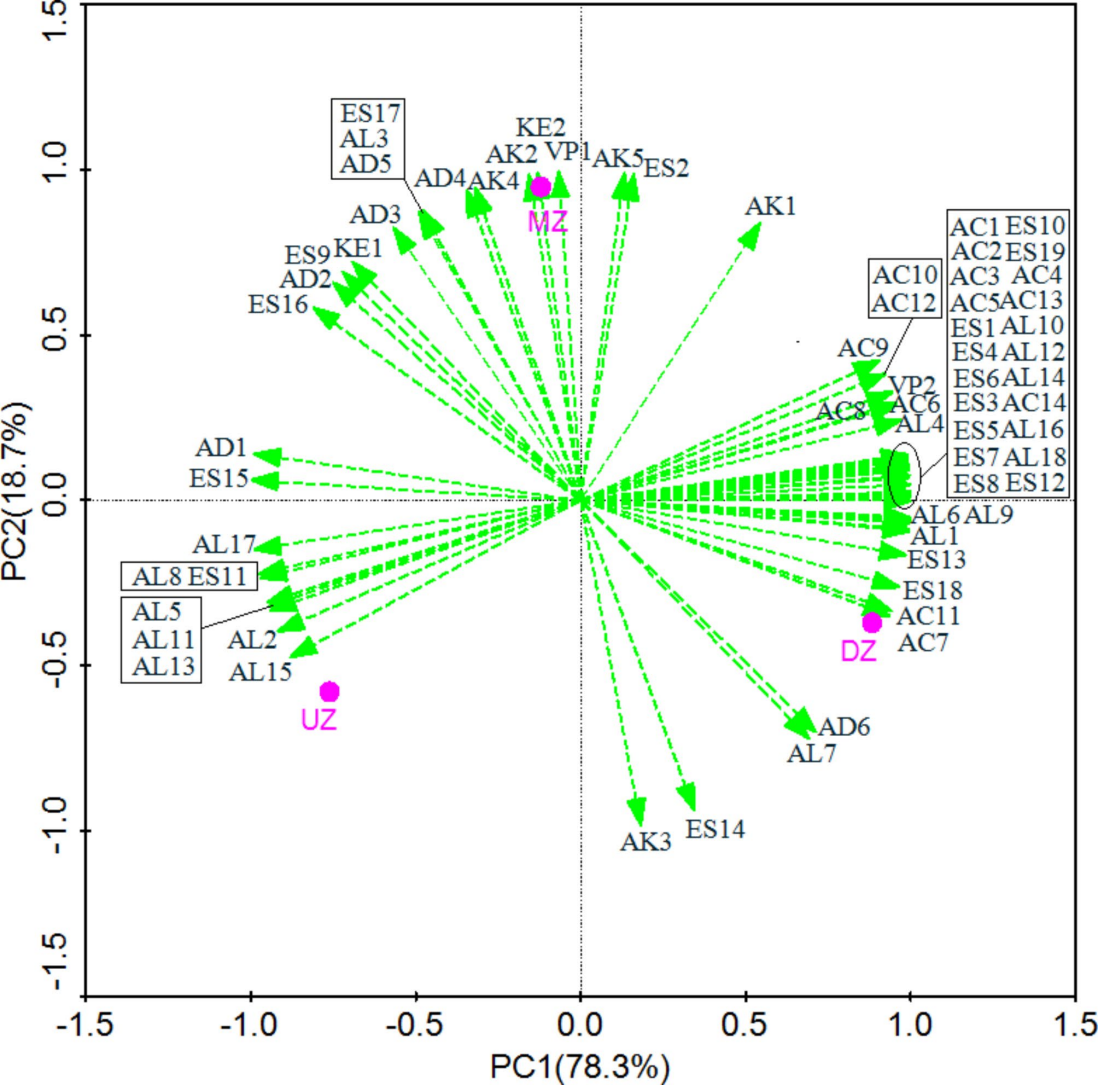
Principal component analysis of volatile compounds on three layers of fermented *Zaopei* samples. The first principal component (X axis) explains 78.3% of the total variance of the dataset, while the second principal component (Y axis) explains 18.7% of the total variance of the dataset. UZ, MZ, and DZ, represent the samples collected from up, middle, and down layer of fermented *Zaopei*, respectively

A PCA approach was next used to assess the distributions of these 66 volatile compounds in different fermented grains sample layers (Fig. 3). Samples from these three layers clearly separated into three clusters based upon the volatile compounds detected therein. The bottom layer of fermented grains contained relatively high levels of volatile acids and esters including acetic acid (AC1), propionic acid (AC2), butyric acid (AC3), caproic acid (AC4), 3-methyl-pentanoic acid (AC5), 2-methyl-butanoic acid (AC6), 2-methyl butanoic acid (AC8), pentanoic acid (AC9), nonanoic acid (AC10), palmitic acid (AC12), octanoic acid (AC13), decanoic acid (AC14), ethyl acetate (ES1), ethyl butanoate (ES3), ethyl hexanoate (ES4), ethyl oenanthate (ES5), ethyl 2-methylbutanoate (ES6), ethyl 3-methylbutanoate (ES7), nonanoic acid ethyl ester (ES8), ethyl heptanoate (ES10), ethyl laurate (ES12), ethyl octadecanoate (ES19), 2-methyl-1-propanol (AL4), 2,3-butanediol (AL10), 2-methylbutanol (AL12), 1-pentanol (AL14), 2-heptanol (AL16), phenylethyl alcohol (AL18), and 2-methoxy-4-vinylphenol (VP2), consisteint with our previous studies demonstrating high levels of esters in this lower fermented grains layer (Yan et al. 2015). In the present analyses, we found that the fusel alcohols isoamyl alcohol (AL2), 1-octen-3-ol (AL5), isooctanol (AL8), octanol (AL11), isopentanol (AL13), 1-nonanol (AL15), and benzyl alcohol (AL17) were primarily concentrated in the upper layer of fermented grains, while the middle fermented grains layer contained high levels of tetradecane (AK2), hexadecane (AK5), ethyl isobutanoat (ES2), ethyl isobutanoat (KE2), phenol (VP1), caryophyllene (AK4), and 2-undecenal (AD4).

### Correlations between yeast communities and volatile compounds

**Fig. 4:**
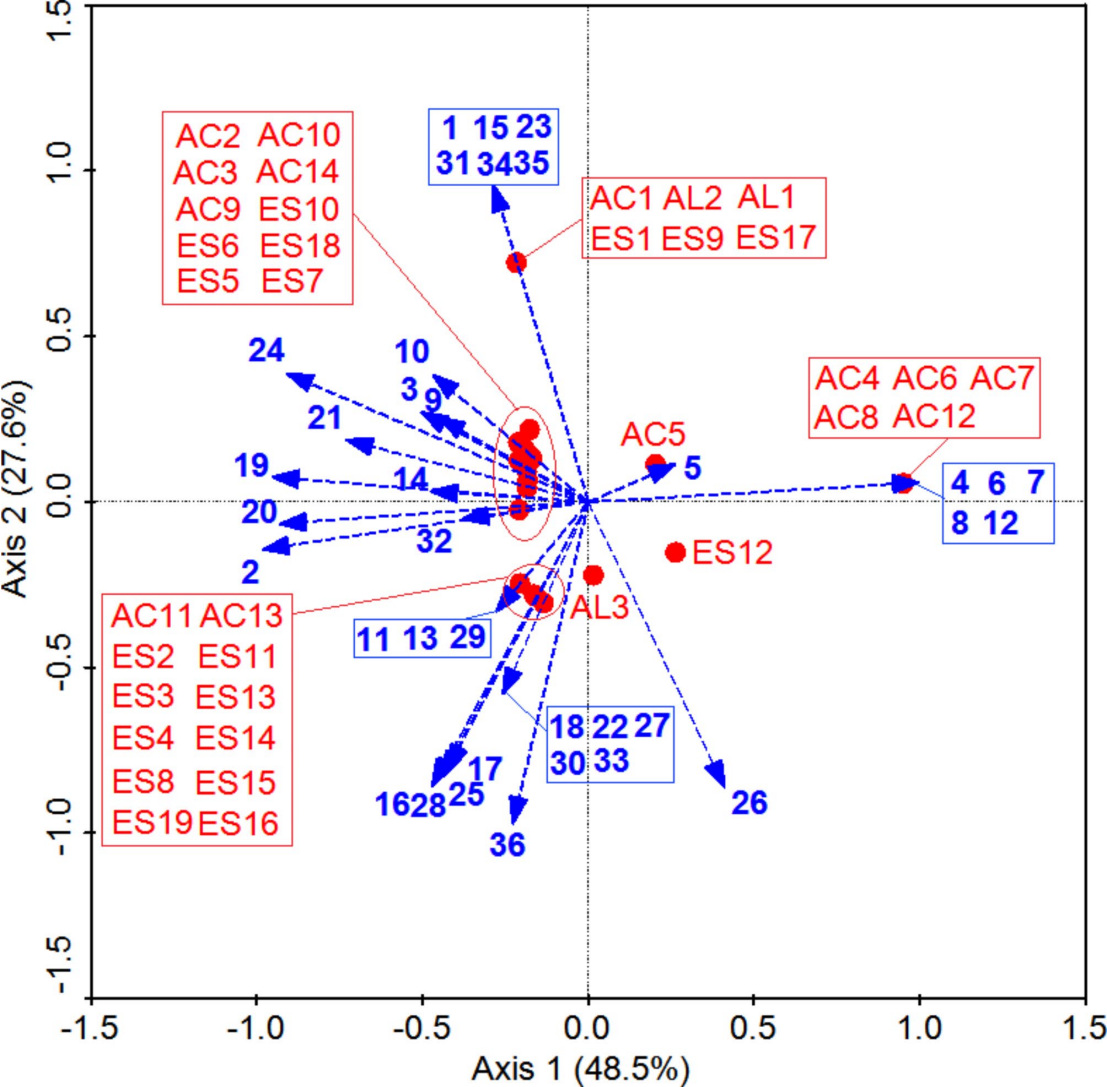
Canonical correspondence analysis (CCA) of yeast community and volatile compounds. Yeasts are numbered as indicated on the DGGE fingerprint files shown in Fig. 2; Table 2

We next conducted a canonical correspondence analysis (CCA) to evaluate correlations between pit mud yeast communities and volatile compounds present in fermented grains. As shown in Fig. 4, the first two component axes in this analysis explained 76.1% of the variation in community composition. *Torulaspora delbrueckii* (4), *Hanseniaspora uvarum* (6), *Saturnispora silvae* (7), *Geotrichum bryndzae* (8), and *Pichia farinosa* (12) were positively correlated with levels of caproic acid (AC4), 2-methyl-butanoic acid (AC6), octanol acid (AC7), 2-methyl butanoic acid (AC8), and palmitic acid (AC12), while *Pichia anomala* (11), *Issatchenkia orientalis* (13), *Yarrowia lipolytica* (16), *Wickerhamomyces anomalus* (17), *Candida intermedia* (18), *Trichosporon asahii* (22), *Pichia guilliermondii* (25), *Candida humilis* (27), *Candida tropicalis* (28), *Cyberlindnera jadinii* (29), *Hanseniaspora vineae* (30), *Metschnikowia spp.* (33), and *Saccharomyces cerevisiae* (36) were positively correlated with levels of hexanoic acid (AC11), octanoic acid (AC13), 1-hexanol (AL3), ethyl butanoate (ES3), ethyl hexanoate (ES4), nonanoic acid ethyl ester (ES8), benzeneacetic acid ethyl ester (ES11), γ-nonylactone (ES13), ethyl oleate (ES14), ethyl pentadecanoate (ES15), ethyl 9-hexadecenoate (ES16), and ethyl octadecanoate (ES19). *Geotrichum silvicola* (2), *Geotrichum silvicola* (3), *Geotrichum bryndzae* (9), *Saccharomycopsis fibuligera* (10), *Alternaria tenuissima* (14), *Pichia kudriavzevii* (19), *Pichia kudriavzevii* (20), *Pichia occidentalis* (21), *Kazachstania barnettii* (24), and *Cryptococcus laurentii* (32) were closely associated with levels of propionic acid (AC2), butyric acid (AC3), pentanoic acid (AC9), nonanoic acid (AC10), decanoic acid (AC14), ethyl oenanthate (ES5), ethyl 2-methylbutanoate (ES6), ethyl 3-methylbutanoate (ES7), ethyl heptanoate (ES10), and ethyl linoleate (ES18). *Geotrichum silvicola* (1), *Candida mucifera* (15), *Trichosporon asahii* (23), *Cryptococcus laurentii* (31), *Rhodotorula dairenensis* (34), and *Saccharomyces cerevisiae* (35) were positively correlated with 3-methyl-butanol (AL1), isoamyl alcohol (AL2), ethyl acetate (ES1), ethyl decanoate (ES9), and ethyl palmitate (ES17) levels.

## Discussion

Distilled liquors contain a high ethanol content, and Chinese liquors are those to be among the oldest distillates in the world (Pu and Yan 2022). Chinese liquors are broadly classified into 12 different flavour types. Of these, strong-flavour liquor is the most popular in China. This liquor is prepared via fermentation in specialized rectangular pit mud cellars (Tan et al. 2020). This pit mud provides an effective habitat for microbial growth and metabolism during liquor distillation, with the microbes therein serving as important determinants of the flavour of the resultant alcohol (Wu et al. 2009). Pit mud composition is thus a key factor influencing Chinese strong-flavour liquor quality (Xu et al. 2017). Pit mud can provide an environment conducive to fermentation, with the filtration and heat retention properties of this material having a pronounced impact on this process. In addition, pit mud can serve as an environment for microbial growth, and the aromatic compounds derived from these microbes can ensure liquor quality. Many microbes are present within pit mud, including bacteria and archaea, and their metabolic byproducts are a primary source of aroma-related compounds (Zhao et al. 2012). As such, most studies of pit mud to date have focused on bacteria.

Although the yeast are an essential part of pit mud microorganisms (Zhao et al. 2012), the diversity of yeast was low, with only the genera *Wickerhamomyces*, *Kluyveromyces*, *Pichia*, *Candida*, Zygosaccharo-myces, and *Geotrichum* was reported in previous investigation (Wang et al. 2017). In the present study, we employed both culture-dependent and PCR-DGGE approaches to facilitate multidimensional analyses the yeast communities of pit mud. Our data suggested that there were significant differences in yeast communities in different pit mud layers. *Geotrichum silvicola* (band 1), *Pichia farinosa* (band 12), *Kazachstania barnettii* (bands 24), *Pichia guilliermondii* (band 25), *Hanseniaspora spp.* (band 26), *Candida humilis* (band 27), *Cyberlindnera jadinii* (band 29), and *Cryptococcus laurentii* (band 32) were only detected in the middle pit mud layer, whereas *Torulaspora delbrueckii* (band 4), *Hanseniaspora uvarum* (band 6), *Candida tropicalis* (band 28), *Hanseniaspora vineae* (band 30), and *Rhodotorula dairenensis* (band 34) were only present within the bottom layer. In addition, *Geotrichum bryndzae* (band 9) and *Issatchenkia orientalis* (band 13) were only present in the bottom pit mud layer. PCA analyses revealed clear differences in the microbial profiles of pit mud samples from different cellar locations (Fig. 3).

In our culture-dependent analysis, we did not detect the presence of several yeast species (*Geotrichum silvicola*, *Torulaspora delbrueckii, Hanseniaspora uvarum*, *Saturnispora silvae, Issatchenkia orientalis*, *Candida mucifera, Kazachstania barnettii*, *Cyberlindnera jadinii, Hanseniaspora spp. Alternaria tenuissima, Cryptococcus laurentii*, *Metschnikowia spp.*, and *Rhodotorula dairenensis*) that were observed via PCR-DGGE. In contrast, other species (*Schizosaccharomyces pombe* and *Debaryomyces hansenii*) were detected only in culture-dependent analyses and not in DGGE fingerprint profiles. These findings emphasize the value of simultaneously conducting both culture-dependent and -independent assays in order to fully characterize pit mud yeast communities. Interestingly, many of the species detected in the present analysis were similar to those detected in our prior study of the microbial communities associated with *Daqu*-starter samples (Yan et al. 2019). Indeed, *Daqu*-starter is generally utilized as a crude microorganism source containing high levels of yeast. *Daqu*-starter accounts for 10–20% of the total raw material used in the liquor production process, suggesting that the microbial community of pit mud is largely influenced by the *Daqu*-starter.

The contents of flavour compounds in fermented grains displayed striking changes associated with the spatial locations of the cellar. And the microbiotas also showed striking changes associated with spatial location. Because the various flavour components are produced by the diversity of micro-organisms in the pit-mud. In our multidimensional HS-SPME-GC-MS analysis, we detected 66 volatile compounds in analyzed fermented grains samples, revealing the highest levels of these volatile acids, esters, and alcohols in the bottom layer of fermented grains, in line with prior studies (Zhang et al. 2020). The middle fermented grains layer contained the highest levels of aldehydes, ketones, alkanes, and volatile phenols, followed by the bottom layer. A CCA approach further revealed strong correlations between pit mud yeast community composition and the volatile flavour compounds detected in fermented grains samples, suggesting that yeast species are likely to have a profound impact on the flavour of Chinese strong-flavour liquor even though they are present at relatively low levels in pit mud as compared to bacterial species (Zhang et al. 2015). It was previously reported that *Saccharomyces cerevisiae* have the ability to ferment saccharides from the fermentative raw materials (cereals) of Chinese strong-flavour liquor to obtain ethanol (Wu et al. 2015). *Candida* and *Pichia* have the ability to metabolize esterases for the biocatalytic synthesis of flavour esters in the liquor (Raghavendra et al. 2014). *Saccharomycopsis fibuligera* produces ethanol and higher alcohols as well as substantial levels of esters and volatile acids(Liu et al. 2017). These results can be also confirmed by our present study.

This study is the first we are aware of to have assessed pit mud yeast community composition via both culture-dependent and –independent approaches. By highlighting the potential importance of yeast as determinants of fermented grains flavour, our results provide a strong foundation for the study and improvement of pit mud composition during Chinese strong-flavour liquor fermentation.

## Electronic supplementary material

Below is the link to the electronic supplementary material.

Supplementary Material 1

## Data Availability

Please contact author for data requests.
