# Supplementary material for: Yeast diversity in pit mud and related volatile compounds in fermented grains of chinese strong-flavour liquor
Source: AMB Express. 2023 Jun 8;13:56. doi: 10.1186/s13568-023-01562-7 (PMC10250287; doi:10.1186/s13568-023-01562-7)
Supplement: Supplementary file 1 — Supplementary Material 1 [file 13568_2023_1562_MOESM1_ESM.doc]

**Supplementary materials**

**Supplementary materials 1: Fig. S1**

(A) (B)

Fig. S1 Cellar and pit mud for the fermentation of Chinese strong-flavour liquor. The bottom and walls of the cellar are constructed with pit mud. (A) Pit mud sampling sites. (B) Fermented *Zaopei* sampling sites.

**Supplementary materials 2: Nucleotide sequences of the bands**

Band 1:

ATTCCCAAACAACTCGACTCGTCGAAGGAGCTTCACACGGGCACGGAACACCCCATCCCAGACGGGACTCTCACCCTCTCCGGCGGCCCGTTCCAGGGCACTTAGACGGGGGCCGCACCCGAAGCATCCTCTGCAAATTACAACTCGGACCCCGACGGGGCCAGATTTCAAATTTGAGCTCTTGCCGCTTCACTCGCCGTTACTGGGGCAATCCCTGTTGGTTTCTTTTCCTCCGCTTATTGATATCGC

Band 2:

ATTCCCAAACAACTCGACTCTTCGAAAGTATCTTACAATGAGAATAGATTTTACGGGGCTGTCACCCTCTAAGGCGCTGTGTTCCAACAGACTTTAATCCGCTCTCAAGATACAATCTACAAATTACAACTCGACCTGGAGGCCGATTTCAAATTTGAGCTTTTGCCGCTTCACTCGCCGTTACTAAGGCAATCCCTGTTGGTTTCTTTTCCTCCGCTTATTGATATCGC

Band 3:

ATTCCCAAACAACTCGACTCGTCGAAGGAGCTTCACACAGGCTTGGTGTCCGACCATACGGGGCTCTCACCCTCTGTGGCGTCCCGTTCCAGGGAACTCGGAAGGCACCGCGCCAGAAGCATCCTCTGCAAATTACAACTCGGGCCGAGGCCAGATTTCAAATTTGAGCTGTTGCCGCTTCACTCGCCGTTACTAGGGCAATCCCTGTTGGTTTCTTTTCCTCCGCTTATTGATATCGC

Band 4:

GCGATATCAATAAGCGGAGGAAAAGAAACCAACCGGGATTGCCTCAGTAACGGCGAGTGAAGCGGCAAGAGCTCAAATTTGAAAGCTGGCTCCTTCGGAGTCCGCATTGTAATTTGCAGAGGATGCTTTGGGTGCGGCTCCCGTCTAAGTGCCCTGGAACGGGCCGTCAGAGAGGGTGAGAATCCCGTCTTGGGCGGGGTGTCCGTGCCCGTGTAAAGCTCCTTCGACGAGTCGAGTTGTTTGGGAAT

Band 5:

GCGATATCAATAAGCGGAGGAAAAGAAACCAACAGGGATTGCCTCAGTAACGGCGAGTGAAGCGGCAACAGCTCAAATTTGAAATCTGGCCTCACGGTCCGAGTTGTAATTTGTAAAGGATGCTTCGAGCATGGTCTGGCCTAAGTTCCTTGGAACAGGACGTCATAGAGGGTGAGAATCCCGTATGCGGCCAGGTGCCTACGCTCATGTGAAGCTCCTTCGACGAGTCGAGTTGTTTGGGAAT

Band 6:

ATTCCCAAACAACTCGACTCGTCGAAGGAGCTTTACACGGGCACGGACACCCCGCCCAAGACGGGATTCTCACCCTCTCTGACGGCCCGTTCCAGGGCACTTAGACGGGGGCCGCACCCAAAGCATCCTCTGCAAATTACAATGCGGACCCCGAGGGGGCCAGCTTTCAAATTTGAGCTCTTGCCGCTTCACTCGCCGTTACTGAGGCAATCCCGGTTGGTTTCTTTTC

CTCCGCTTATTGATATCGC

Band 7:

ATTCCCAACAACTCGACTCTTCGAGAGCGCATCACAGAAGCACAGGCATCCGTGCCAAAGACGGGATTCTCACCCTCTATGACGCTCCATTCCAGGAGACTTGTGCACGGCCCGGCGCGGAAGACGCTTCTCTAAATTACAACTCGGACGGCCAGAGACCGCCAGATTTTAAATTTGAGCTTTTCCCGCTTCACTCGCAGTACTAGGGGAATCCTTGTTAGTTTCTTTTCCTCCGCTTATTGATATCGC

Band 8:

ATTCCCAAACAACTCGACTCGTCGAAGGAGCTTCACACGGGCACGGAACACCCCATCCCAGACGGGATTCTCACCCTCTCCGATGGCCCATTCCAGGGCACTTAGACAGGGGCCGCACCCGAAGCATCCTCCACAAATTACAACTCGGACCCCGGAGGGGCCAGATTTCAAATTTGAGCTCTTGCCGCTTCACTCGCCGTTACTAGGGGAATCCTTGTTAGTTTCTTTTCCTCCGCTTATTGATATCGC

Band 9:

ATTCCCAAACAACTCGACTCGTCGAAGGAGCTTCACACAGGCTAGGCGCACGACCGGACGGGGCTCTCACCCTCTGTGGCGTCCCGTTCCAGGGAACTCGGAAGGCGCGTCGCCAGAAGCATCCTCTGCAAATTACAACTCGGGCCGGGGGGCCAGATTTCAAATTTGAGCTGTTGCCGCTTCACTCGCCGTTACTGGGGCAATCCCTGTTGGTTTCTTTTCCTCCGCTTATTGATATCGC

Band 10:

ATTCCCAAACAACTCGACTCGTCGAAGGAGCTTCACACGGGCGCGGCCACCCCATCCCAGACGGGATTCTCACCCTCTATGACGGCCCGTTCCAGGGCACTTAGATGGGGGCCGTTCCCGAAGCATCCTCTGCAAATTACAATGCGGACCCCGAGGGGGCCAGCTTTCAAATTTGAGCTCTTGCCGCTTCACTCGCCGTTACTAGGGCAATCCCGGTTGGTTTCTTTTCCTCCGCTTATTGATATCGC

Band 11:

ATTCCCAAACAACTCGACTCGTTGACAGCGCCTCGTGGTGCGACAGGGTCCGAGCGCAACGGGGCACTCACCCTCTCTGGCGCCCCCTTCCAGGGGACTTGTGCCCGGTCCGTCGCTGAGGACGCTTCTCCAGACTACAATTCGAACGCCGAGGGCGCCCGATTCTCAAGCTGGGCTCTTCCCGGTTCGCTCGCCGTTACTAAGGGAATCCTTGTAAGTTTCTTTTCCTCCGCTTATTGATATCGC

Band 12:

ATTCCCAAACAACTCGACTCGTAGAAGACGTATCACAGAGCACCGGTGGTCGTGTTAAGTACGGGATTATCACCCTCTTTGATACTCCTTTCCAGGAGACTTGGACACGGTCCGGCACGGAAAACGCCTCTATAGATTACAACTCGGACAATCGAAGACTGCCAGATTTCAAATTTGAGCTCTTCCCGCTTCACTCGCCGTTACTAGGGGAATCCTTGTTAGTTTCTTTTCCTCCGCTTATTGATATCGC

Band 13:

ATTCCCAAACAACTCGACTCGTCGAAGGAGCTTTACACAGGCTAGGCGTCCGACCAGACGGGGCTCTCACCCTCTATGGCGTCTCGTTCCAGGGAACTCGGAAGGCACCGCGCCAAAAGCATCCTCTGCAAATTACAACTCGGGCCCTGGGGGCCAGATTTCAAATTTGAGCTGTTGCCGCTTCACTCGCCGTTACTGGGGCAATCCCTGTTGGTTTCTTTTCCTCCGCTTATTGATATCGC

Band 14:

ATTCCCAAACAACTCGACTCTTGGAGCATGCATCGCAATAGACTCTTTCAAGACCCTCTACGGGATTGTCACCCTCTGTGAAGCCTTATTCCAAAGGACTTGAAGGTCCAAAGAGCCTGGATAGCACGTCCTTAGTTTACAATCCGGTTTGTCTAGGACAAACAGGTTTTAAACTTGAGCTTTACCCACTTCGCTCGCCGCTACTGAGGGTATCATTGTTATTTTCTTTTCCTCCGCTTATTGATATCGC

Band 15:

ATTCCCAAACAACTCGACTCGTCGAAGGAGCTTCACACGGACGCGGCCACCCGTCCCAGACGGGACTCTCACCCTCTCCGGCGGCCCGTTCCAGGGCACTTAGACGGGGGCCGCACCCGAAGCATCCTCTGCAAATTACAACTCGGACCCCGACGGGGCCAGATTTCAAATTTGAGCTCTTGCCGCTTCACTCGCCGTTACTGGGGCAATCCCTGTTGGTTTCTTTTCCTCCGCTTATTGATATCGC

Band 16:

ATTCCCAAACAACTCGACTCTTCGAAAGTATCTTACAATGAGAATAGATTTTACGGGGCTGTCACCCTCTAAGGCGCTGTGTTCCAACAGACTTTAATCCGCTCTCAAGATACAATCTACAAATTACAACTCGACCTGGGGGCCGATTTCAAATTTGAGCTTTTGCCGCTTCACTCGCCGTTACTAAGGCAATCCCTGTTGGTTTCTTTTCCTCCGCTATTGATATCGC

Band 17:

ATTCCCAAACAACTCGACTCGTCGGAAGACAAACTGAATAACTGCTAGTCAACTCACGGGGCTCTCACCCTCTACGGCGGCCTATTCCAAGGCACTTAGTTGACACAGCCACCCAAATAGTCTACCTCCAAATTACAACTCGGGCTTACGCCWGATTTCAAATTTGGGCTGTTGCCGCTTCGTTCGCCATTACTGAGGCAATCCCTGTTGGTTTCTTTTCCTCCGCTTATTGAATATCGC

Band 18:

ATTCCCAACAACTCGACTCGTCGAAGGAGCTTCACACGGGCGCGGACACCCCATCCCATACGGGATTCTCACCCTCTATGACGTCCCGTTCCAGGGCACTTAGATGGGGACCGCTCCCGAAGCATCCTCTACAAATTACAATGCGGACCCCGAAGGAGCCAGCTTTCAAATTTGAGCTYTTGCCGCTTCACTCGCCGTTACTRRGGCAATCCCTGTTGGTTTCTTTTCCTCCGCTTATTGATATCGC

Band 19:

ATTCCCAAACAACTCGACTCGTTGAAGGAGCTTCACACGGGCACGGGGCTCCCCGTCCCAGACGGGATTCTCACCCTCTCCGATGGCCCGTTCCAGGGCACTTAGACAGGGGCCGCACCCGAAGCATCCTCTACAAATTACAACTCGGACCCCGGAGGGGCCAGATTTCAAATTTGAGCTCTTGCCGCTTCACTCGCCGTTACTGAGGCAATCCCGGTTGGTTTCTTTTCCTCCGCTTATTGATATCGC

Band 20:

ATTCCCAAACAACTCGACTCGCAGACAGCGCCTCGTGGTGCAACAGGGTCCGGGCACGACGGGGCTCTCACCCTCTCTGGCGCCCCCTTCCAGGGGACTTGGGCCCGGTCCGCCGCTGAGGACGCTTCTCCAGACTACAATTCGGACGACGAAGCCGACCGATTTTCAAGCTGGGCTGTTCCCGGTTCGCTCGCCGTTACTAAGGGAATCCTTGTAAGTTTCTTTTCCTCCGCTTATTGATATCGC

Band 21:

ATTCCCAAACAACTCGACTCTTCGAAAGTATCTTACAATGAGAATAGATTTTACGGGGCTGTCACCCTCTAAGGCGCTGTGTTCCAACAGACTTTAATCCGCTCTCAAGATACAATCTACAAATTACAACTCGACCTGGTGGCCGATTTCAAATTTGAGCTTTTGCCGCTTCACTCGCCGTTACTAAGGCAATCCCTGTTGGTTTCTTTTCCTCCGCTTATTGATATCGC

Band 22:

ATTCCCAAACAACTCGACTCGTCGAAGGAGTTTCACAGAGGCTTAGCGACCAACCGTACGGGGCTCTCACCCTCTATGGCGTCCCGTTCCAGGGAACTCGGAAGGCACCTCGCCAGAAACATCCTCTGCAAATTACAACTCGGGCCTAGGGCCAGATTTCAAATTTGAGCTGTTGCCGCTTCACTCGCCGTTACTGAGGCAATCCCTGTTGGTTTCTTTTCCTCCGCTTATTGATATCGC

Band 23:

ATTCCCAAACAACTCGACTCGTCGAAGGAGTTTCACAGAAGGCTTAGCGACCAACCGTACGGGGCTCTCACCCTCTATGGCGTCCCGTTCCAGGGAACTCGGAAGGCACCTCGCCAGAAACATCCTCTGCAAATTACAACTCGGGCCTAGGGCCAGATTTCAAATTTGAGCTGTTGCCGCTTCACTCGCCGTTACTGAGGCAATCCCTGTTGGTCTCTTTTCCTCCGCTTATTGATATCGC

Band 24:

ATTCCCAAACAACTCGACTCGTCGAAGGAGCTTCACATGGACGTGGACACCCCGTCCCAGACGGGATTCTCACCCTCTCCGATGGCCCGTTCCAGGGCACTTAGACGGGGGCCGCACCCGAAGCATCCTCTACAAATTACAACTCGGACCCCGGAGGGGCCAGATTTCAAATTTGAGCTCTTGCCGCTTCACTCGCCGTTACTGAGGCAATCCCGGTTGGTTTCTTTTCCTCCGCTTATTGATATCGC

Band 25:

ATTCCCAAACAACTCGACTCGTCGAAGGAGCTCCACATGAGCGTAGGCACCTGGCCGCATACGGGATTCTCACCCTCTATGACGTCCTGTTCCAAGGAACTTAGGCCAGACCATGCTCGAAGCATCCTCTACAAATTACAACTCGGACCGTGAGGCCAGATTTCAAATTTGAGCTGTTGCCGCTTCACTCGCCGTTACTGAGGCAATCCCTGTTGGTTTCTTTTCCTCCGCTTATTGATATCGC

Band 26:

ATTCCCAAACAACTCGACTCGTCGAAGGAGCCTCACATGGACGCGGACACCCCGTCCCAGACGGGATTCTCACCCTCTCCGATGGCCCGTTCCAGGGCACTTAGACGGGGGCCGCACCCGAAGCATCCTCTACAAATTACAACTCGGACCCCGGAGGGGCCAGATTTCAAATTTGAGCTCTTGCCGCTTCACTCGCCGTTACTGAGGCAATCCCGGTTGGTTCCTTTTCCTCCGCTTATTGATATCGC

Band 27:

ATTCCCAAACAACTCGACTCGTCGAAGGAGCTTCACACGGGCGCGGACACCCCATCCCATACGGGATTCTCACCCTCTATGACGTCCCGTTCCAGGGCACTTAGATGGGGACCGCTCCCGAAGCATCCTCTACAAATTACAATGCGGACCCCGAAGGAGCCAGCCTTCAAATTTGAGCTCTTGCCGCTTCACTCGCCGTTACTGGGGCAATCCCTGTTGGTTTCTTTTCCTCCGCTTATTGATATCGC

Band 28:

ATTCCCAAACAACTCGACTCTTAGAGCGTGTATCACAAAGCACGGTACATCCATGGCAAGTACGGGATTTTCACCCTCTACGATGCCCTGTTCCAAGGGACTTGTCCATAGAGCCGCACGGAAAACACGTCTCGAGATTACAACGCGGACACCGGAGGTGCCAGCTTTCAAATTTGAGCTCTTCCCGCTTCGCTCGCCGTTACTAGGGGAATCCTTGTTAGTTTCTTTTCCTCCGCTTATTGATATCGC

Band 29:

ATTCCCAAACAACTCGACTCGTCGAAGGAGCTTCACATGAGCGTAGGCACCTGGCCGCATACGGGATTCTCACCCTCTATGACGTCCTGTTCCAAGGAACTTAGGCCAGACCATGCTCGAAGCATCCTCTACAAATTACAACTCGGACCGTGAGGCCAGATTTCAAATTTGAGCTGTTGCCGCTTCGCTCGCCGTTACTGAGGCAATCCCTGTTGGTTTCTTTTCCTCCGCTTATTGATATCGC

Band 30:

ATTCCCAAACAACTCGACTCGTCGAAGGAGCTTTACACGGGCACGGACACCCCGCCCAAGACGGGATTCTCACCCTCTCTGACGGCCCGTTCCAGGGAACTTAGACGGGGGCCGCACCCAAAGCATCCTCTGCAAATTACAATGCGGACTCCGAAGGAGCCAGCTTTCAAATTTGAGCTCTTGCCGCTTCACTCGCCGTTACTGAGGCTATCCCGGTTGGTTTCTTTTCCTCCGCTTATTGATATCGC

Band 31:

ATTCCCAAACAACTCGACTCGTCGAAGGAGCTTCACACGGGCGCGGACACCCCATCCCATACGGGATTCTCACCCTCTATGACGTCCCGTTCCAGGGCACTTAGATGGGGACCGCTCCCGAAGCATCCTCTACAAATTACAATGCAGACCCCGAAGGAGCCAGCTTTCAAATTTGAGCTCTTGCCGCTTCACTCGCCGTTACTGGGGCAATCCCTGTTGGTTTCTTTTCCTCCGCTTATTGATATCGC

Band 32:

ATTCCCAAACAACTCGACTCGTCGAAGGAGCTTCACACGGGCGCGGACACCCCATCCCATACGGGATTCTCACCCTCTATGACGTCCCGTTCCAGGGCACTTAGATGGGGACCGCTCCCGAAGCATCCTCTACAAATTACAATGCGGACCCCGAAGGAGCCAGCTTTCAAATTTGAGCTCTTGCCGCTTCACTCGCCGTTACTGGGGCAATCCCTGTTGGTTTCTTTTCCTCCGCTTATTGATATCGC

Band 33:

ATTCCCAAACAACTCGACTCGTCGAAGGAGCTTTACACGGGCACGGACACCCCGCCCAAGACGGGATTCTCACCCTCTCTGACGGCCCGTTCCAGGGCACTTAGACGGGGGCCGCACCCAAAGCATCCTCTGCAAATTACAATGCGGACTCCGAAGGAGCCAGCTTTCAAATTTGAGCTCTTGCCGCTTCACTCGCCGTTACTGAGGCAATCCCGGTTGGTTTCTTTTCCTCCGCTTATTGATATCGC

Band 34:

ATTCCCAAACAACTCGACTCTTAGAGCGTGTATCACAAAGCACGGTACATCCATGGCAAGTACGGGATTTTCACCCTCTACGATGCCCCGTTCCAAGGGACTTGTCCACAGAGCCGCACGGAAAACACGTCTCGAGATTACAACGTGGACACCGGAGGTGCCAGCTTTCAAATTTGAGTTCTTCCCGCTTCGCTCGCCGTTACTAGGGGAATCCTTGTTAGTTTCTTTTCCTCCGCTTATTGATATCGC

Band 35:

ATTCCCAAACAACTCGACTCGTCGAAGGAGCTTCACATGAGCGTAGGCACCTGGCCGCATACGGGATTCTCACCCTCTATGACGTCCTGTTCCAAGGAACTTAAGCCAGACCATGCTCGAAGCATCCTCTACAAATTACAACTCGGACCGTGAGGCCAGATTTCAAATTTGAGCTGTTGCCGCTTCACTCGCCGTTATTGAGGCAATCCCTGTTGGTTTCTTTTCCTCCGCTTATTGATATCGC

Band 36:

ATTCCCAAACAACTCGACTCCTAGAGCGTGTATCACAAAGCACGGTACATCCATGGCAAGTACGGGATTTTCACCCTCTACGATGCCCTGTTCCAAGGGACTTGTCCATAGAGCCGCACGGAAAACACGTCTCGAGATTACAACGCGGACACCGGAGGTGCCAGCTTTCAAATTTGAGCTCTTCCCGCTTCGCTCGCCGTTACTAGGGGAATCCTTGTTAGTTTCTTTTCCTCCGCTTATTGATATCGC
